# New technology to assess sleep apnea: wearables, smartphones, and accessories

**DOI:** 10.12688/f1000research.13010.1

**Published:** 2018-03-29

**Authors:** Thomas Penzel, Christoph Schöbel, Ingo Fietze

**Affiliations:** 1 Interdisciplinary Sleep medicine Center, Charité – Universitätsmedizin Berlin, Freie Universität Berlin, Humboldt-Universität zu Berlin, and Berlin Institute of Health, Berlin, Germany; 2 International Clinical Research Center, St. Anne's University Hospital Brno, Brno, Czech Republic

**Keywords:** Sleep apnea, sleeping disorder, polysomnography

## Abstract

Sleep medicine has been an expanding discipline during the last few decades. The prevalence of sleep disorders is increasing, and sleep centers are expanding in hospitals and in the private care environment to meet the demands. Sleep medicine has evidence-based guidelines for the diagnosis and treatment of sleep disorders. However, the number of sleep centers and caregivers in this area is not sufficient. Many new methods for recording sleep and diagnosing sleep disorders have been developed. Many sleep disorders are chronic conditions and require continuous treatment and monitoring of therapy success. Cost-efficient technologies for the initial diagnosis and for follow-up monitoring of treatment are important. It is precisely here that telemedicine technologies can meet the demands of diagnosis and therapy follow-up studies. Wireless recording of sleep and related biosignals allows diagnostic tools and therapy follow-up to be widely and remotely available. Moreover, sleep research requires new technologies to investigate underlying mechanisms in the regulation of sleep in order to better understand the pathophysiology of sleep disorders. Home recording and non-obtrusive recording over extended periods of time with telemedicine methods support this research. Telemedicine allows recording with little subject interference under normal and experimental life conditions.

## Introduction

Sleep medicine is gaining importance because more and more people focus their attention on sleep complaints and sleep disorders. Not all complaints about sleep problems require immediate attention by a sleep physician with diagnostic procedures and therapeutic interventions. However, good information for primary care physicians can help them decide which complaint requires more attention and which complaint can be handled with information and behavioral advice. Overall, the discipline of sleep medicine is growing and the number of sleep experts and sleep physicians is increasing worldwide. The number of sleep centers is increasing as well, albeit at a slower pace.

The prevalence of most sleep disorders has been increasing recently. The prevalence of sleep-related breathing disorders, among all sleep disorders, has raised the most attention. A careful review of the prevalence of sleep-related breathing disorders showed that the prevalence of sleep apnea has increased over the last two decades ^1^. In 1993, the first systematic study of the prevalence for sleep-disordered breathing reported prevalences of 4% in men and 2% in women ^2^. A recent meta-analysis by Peppard *et al*. showed that 10% of men and 3% of women between the ages of 30 and 49 years and 17% of men and 9% of women between the ages of 50 and 70 years have obstructive sleep apnea (apnea hypopnea index [AHI] ≥ 15/hour) ^1^. This meta-analysis shows an increase in prevalence over 20 years and in addition an increase with the age of the subjects. A large epidemiological cohort study in Switzerland showed prevalences of 49% in men and 23% in women at an age of over 40 years on the basis of an AHI of at least 15/hour ^3^. In the study by Heinzer, not all subjects reported subjective complaints of sleepiness, even if this is an often-reported consequence of moderate sleep apnea (AHI of at least 15/hour). From the results of this epidemiological study, it becomes obvious that only a subgroup of subjects with an AHI of at least 15/hour seek medical help from a sleep physician. As a consequence, diagnostically relevant sleep apnea without clear symptoms remains undiagnosed and untreated in many cases. Whether those persons with sleep apnea without symptoms and not seeking sleep medicine help have health consequences needs to be investigated in future epidemiological studies.

## Diagnostic procedures for sleep apnea

The diagnostic procedure in sleep medicine begins with a sleep-focused clinical interview followed by a review of clinical findings and the results of a home sleep study and of an attended polysomnography sleep study if applicable. The final result of the diagnostic procedure is a diagnosis according to the *International Classification of Sleep Disorders*, third edition (ICSD-3) ^4^. This diagnostic manual distinguishes six groups of sleep disorders and a total of 66 sleep medicine disorders. The diagnostic groups are insomnia, sleep-related breathing disorders, hypersomnias of central origin, parasomnias, sleep-related movement disorders, and disorders of the sleep-wake rhythm. Besides these six categories, there is a seventh category for secondary sleep disorders which may be the consequence of medical problems, mental problems, or environmental problems (for example, light, noise, and inappropriate sleep environment). An eighth category summarizes unspecific disorders not otherwise classified. These are normal variants such as long sleeper, short sleeper, and snoring, for which the pathological value is not proven.

The core of sleep medicine diagnosis is a certified sleep center. A sleep center can perform a sleep study with attended polysomnography. Attended polysomnography represents the reference method for the quantitative study of sleep. Even if home sleep studies become more common, these studies usually record a small fraction of signals and information compared with polysomnography. However, attended polysomnography remains the diagnostic reference ^5^. According to the recommendations of the American Academy of Sleep Medicine, such a polysomnography comprises three electroencephalography (EEG) leads, two electrooculography (EOG) leads, and three electromyography (EMG) leads for the m. submentalis and the m. tibialis of both legs. The EEG, EOG, and EMG of the m. submentalis are used for the determination of sleep stages. The EMG leads of the two legs are used to diagnose sleep-related movement disorders. A single-channel electrocardiography (ECG) records heart rate to document changes in heart rate variability and to alert for possible sleep-related cardiac arrhythmias. The recording of respiration during sleep requires a nasal pressure sensor (nasal prongs) for airflow, a thermistor as a complementary method for airflow, and two belts with respiratory inductive plethysmography (RIP) for thoracic and abdominal respiratory movements. The effect of respiration is recorded by pulse oximetry for oxygen saturation and pulse rate. If periods of hypoventilation need to be recognized, then carbon dioxide (CO _2_) monitoring is needed as well. In order to do this, a sensor can be used to record transcutaneous CO _2_ partial pressure (tcPCO _2_) continuously. If this expensive sensor system is not available, alternatively one may record end-tidal CO _2_ (ETCO _2_) in the expired airflow. Different devices and sensor systems are available for both technologies. For additional cardiovascular changes, it is most desired to record blood pressure non-invasively and continuously. The best available method is finger photoplethysmography. However, owing to the pressure-applying sensors at the finger, this method is expensive and not comfortable for the patient. In the last few years, pulse transit time has been used to estimate a continuous signal of blood pressure if calibrated against a regular cuff-based blood pressure assessment at the beginning of the recording ^6^. A microphone attached to the trachea is used to document snoring and other noises during sleep, such as speaking. The recording of body position is used for sleep disorders which are position dependent. Video recording is part of attended cardiorespiratory polysomnography. The video is important to document the behavioral changes during sleep. Sleep itself depends heavily on behavior. A person must decide to go to bed and to be ready to fall asleep. During sleep, the sleeper is unconscious. Some sleep disorders like sleep walking happen during sleep and the sleeper is unconscious of this behavior. Video recording helps to recognize these sleep disorders. Sleep physicians diagnose parasomnias and rapid-eye-movement sleep-related disorders during sleep based on the combination of sleep EEG recording and video recording.

## Sleep apnea pathophysiology

Sleep apnea is characterized by cessations of respiration during sleep. The pathomechanism of sleep-disordered breathing is an obstruction of the upper airways in the case of obstructive sleep apnea, whereas in the case of central sleep apnea there is no obstruction of the upper airways. If the airflow does not cease completely, hypopneas are scored. The minimal event duration in adults is 10 seconds, and the typical event duration varies between 30 and 60 seconds. Single events could become as long as 120 seconds or more. An AHI of 15 events per hour corresponds to 15 apnea and hypopnea events per hour of sleep time. Apnea and hypopnea events are accompanied by oxygen desaturation. Usually, only the lowest oxygen saturation value is noted. In aggregated data, an oxygen desaturation index (ODI) is specified. The ODI simply counts the number of desaturations per hour of sleep if a single event presents a desaturation of at least 3% or 4% (depending on written criteria). No more details of oxygen saturation changes have been considered until now, and this is recognized as a weakness in current aggregated sleep reports. Therefore, in more recent analysis methods, the duration of oxygen desaturations and the area under the curve of a particular desaturation event are considered to quantify the severity of sleep-disordered breathing ^7, 8^. This may be a much more comprehensive way, and one that is closer to pathophysiology, to characterize the severity of the disorder. Usually, apnea and hypopnea events are terminated by a central nervous activation called arousal. EEG during polysomnography allows one to record and score arousal ^5^. The multiple arousals disturb sleep by fragmenting it, and this leads to nonrestorative sleep and therefore to excessive daytime sleepiness. However, sleep EEG is not recorded by home sleep apnea testing (HSAT) and therefore these systems do not allow the direct detection of arousals. Usually, the central nervous activation (arousal) at the end of an apnea event with parallel hypoxia does cause an increase in sympathetic tone as well. This is called autonomous nervous system activation. A direct reflection of this autonomous nervous system activation is an increase in heart rate, peripheral vasoconstriction, and an increase in blood pressure. These effects of ending an apnea event are directly responsible for an increased cardiovascular risk. However, none of these parameters has ever been set in relation to severity of sleep apnea or to cardiovascular consequences of sleep apnea. From a pathophysiological point of view, these activations can have serious cardiovascular consequences such as the development of hypertension, cardiac arrhythmias, and an increased risk of myocardial infarction and stroke.

## Recent challenges to sleep apnea management

Epidemiological studies show that the prevalence of sleep-related breathing disorders (based on the definition of an elevated AHI) is much higher than previously assumed. Among these subjects, there are without a doubt many undiagnosed patients. Some of these may even have severe sleep apnea (AHI ≥ 30/hour), and these patients certainly have increased associated health risks. How far the AHI correlates with an increase cardiovascular risk remains an open question.

Earlier studies showed a high cardiovascular risk in patients with severe sleep apnea (AHI of at least 30/hour) compared with control subjects, snorers, and treated sleep apnea patients ^9^. If patients were treated with continuous positive airway pressure (CPAP) just on the basis of the finding of an increased AHI together with a previously existing cardiovascular risk, then no positive effect on mortality and hospital admissions could be found. This is the result of a recent large randomized prospective study ^10^. In that study, after 3.7 years of treatment, no positive effects on cardiovascular mortality and morbidity were found. CPAP therapy reduced snoring and observed daytime sleepiness and improved subjective quality of life. These effects are definitely positive. The cardiovascular risk needs to be reduced in patients identified in the earlier studies as risk sufferers. However, the optimal parameter to identify these patients does not appear to be the AHI. What the best parameters would be, in order to identify the population that has sleep apnea and an increased cardiovascular risk, is still open for future research ^11^.

## Home sleep apnea testing

The high prevalence of sleep apnea was an early trigger of the development and application of portable equipment to allow HSAT. HSAT focuses on the most important variables. These are a distinction of sleep and wakefulness, oronasal airflow, respiratory effort, oxygen saturation, and body position. Even if cardiorespiratory polysomnography remains the reference standard for diagnostic procedures, new generations of portable sleep apnea testing devices have become increasingly accurate with better sensitivity and better specificity. Today, these are the first-line diagnostic methods for establishing the diagnosis of sleep-disordered breathing ^12^. Many different systems are available and have been validated against polysomnography ^13^. In order for the accuracy to be quantitatively assessed, a high sensitivity and a high specificity are essential ^14^. Today’s systems allow the application of sensors by patients themselves at home ^15^. The sleep physician or the sleep nurse just needs to explain the handling of the system when the patient comes in for a consultation and then the patient can perform the recording themselves at home. The next morning, the patient returns the equipment to the outpatient department, and the recorded data are downloaded from the device and are scored by the sleep technician in order to check for sleep time, respiratory events, and positional effects on breathing during the night. The scoring process begins with an automated pre-scoring, and the result is manually edited and corrected when needed. The better the automated pre-scoring is, the less effort needs to be taken to remove artifacts and to correct the scoring results. Once the manual re-scoring is finished, a summary report with aggregated data is presented, a therapeutic decision can be made, and the results are filed for future control studies. This is required because sleep-disordered breathing presents a chronic disorder that requires regular check-ups to test whether the condition has become worse over the years.

Some HSAT systems allow patients to enter sleep times (lights off for intended sleep onset and lights on for intended wake-up time) manually. This will allow the evaluation software to adjust the calculation of indices of respiratory events for corrected sleep duration. Some other systems assess sleep times indirectly by an additional light or activity monitoring. All of these portable systems are called HSAT or polygraphy (PG) in Europe ^16^. Alternative names are OOC (“out-of-center”) recording or portable sleep apnea monitoring ^13^. Continuous online monitoring, as for polysomnography or telemonitoring, is available for some systems but generally is not possible and not needed.

In order to evaluate and categorize systems available for home sleep apnea monitoring, a new scheme has been introduced. The scheme is based on an assessment of the monitored physiological functions. This scheme is called the SCOPER scheme. SCOPER is an acronym for the functions required to be monitored ^13^. The necessary functions required for HSAT have to be assessed with adequate quality and need to be evaluated regarding the parameters required. The functions comprise sleep (S), cardiovascular system (C), oxygen saturation (O), body position (P), respiratory effort (E), and airflow (R) in terms of sensing and recording. Detailed criteria for particular sensor specifications are given. The technologies and technical specifications for the signal recording are specified as well. This means that a microphone, as an example for one particular sensor, is evaluated in terms of detecting respiratory effort (E) and airflow (R) related to the true reference signals such as esophageal pressure and quantitative airflow. This means that the evaluation and categorization of systems are no longer a simple counting of signals (for example, one or two belts for recording respiratory effort with RIP) but instead a checking of the functions needed to characterize sleep-disordered breathing. The assessment of respiratory effort is evaluated by checking whether a system, regardless of sensor type and number, is able to distinguish between obstructive and central apnea and hypopnea events. With this new evaluation scheme, new technologies, blended sensors, and software developments have a chance to fulfill the criteria for the successful diagnosis of sleep-disordered breathing. Innovative technologies, like calculation of respiration derived from an ECG recording or contact-free recording of movement, respiration, and heartbeat using radar frequency technologies, have a chance to recognize apnea and hypopnea events and can be evaluated on the basis of this scheme. They also need to assess sleep and body position in order to fulfill the (S) and (P) requirements. Finally, when reporting, they need to make an evaluation of the severity of the sleep-disordered breathing disorder to be diagnosed.

## Telemedicine methods in sleep medicine defined

The telemedicine applications available are found to a smaller extent in the diagnostic procedures and to a much greater extent in the monitoring of therapy compliance for sleep apnea treatment ^17^. Thus, the area of telemedicine in sleep medicine is often regarded as a very focused and limited application field for telemedicine technologies ^18^, which covers only a very small part of the broad definition for “telemedicine” as “delivering medicine over a distance”. Here, we report on those aspects of telemedicine which are relevant for sleep medicine. Telemedicine is regarded as a method and as a tool being applied in medicine. The applications include the wireless recording of sleep and vital functions during sleep, wireless recording of respiration, wireless data transmission for recorded data, assessment of sleep and sleep apnea using smartphone technologies, and telemedicine monitoring of therapy compliance in treated patients with sleep apnea ^19^. Smartphone applications are briefly reviewed as well, and the smartphone is seen as another tool in diagnostic and therapeutic procedures. Also, consumer products, usually smartphone- or tablet-based, to track or modify sleep are becoming widely available ^20^. These applications are briefly mentioned in the sections below; however, for more in-depth discussions, we refer the reader to the excellent systematic overview by Ko *et al*. ^20^.

## Diagnostic telemedicine applications

**Figure 1.**
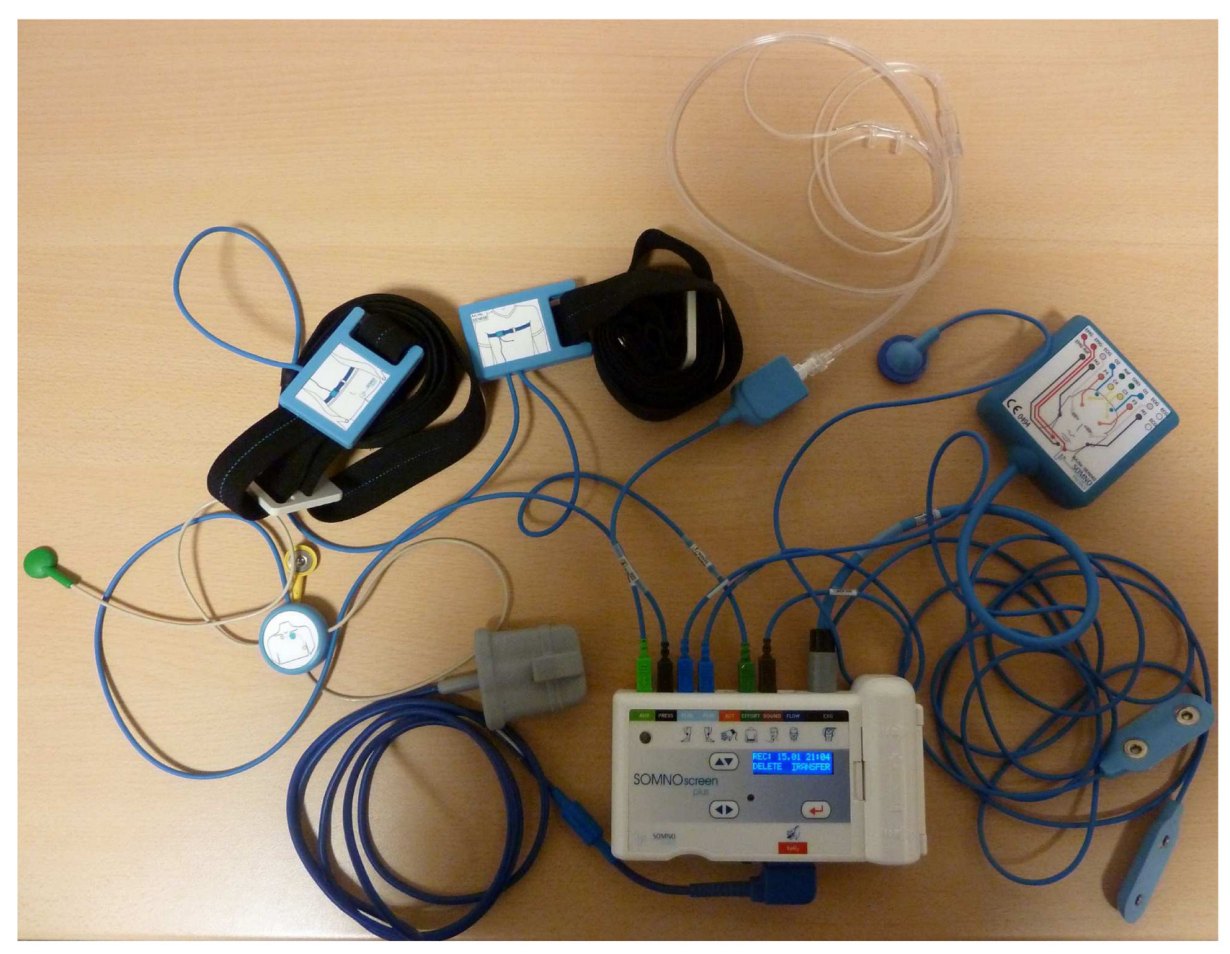
Polysomnography system with telemedicine options. The image shows a system configurable for sleep medicine diagnosis. This particular system can be used for attended polysomnography in a sleep laboratory with all signals to be recorded. Data are stored on a flash memory or are transmitted using a wireless network (or a wired network if required). The system can be set up as attended polysomnography, as home polysomnography, or as home sleep apnea monitoring, depending on the number of sensors and the configuration chosen.

Many modern diagnostic systems can be configured to be used as a polysomnography system or as a PG system for HSAT ( Figure 1). Devices available today offer a wireless transmission of recording modules (for example, for oxygen saturation on the finger) to the main body unit worn at the chest (by Bluetooth technology). This wireless connection of modules with a main unit is called body area network (BAN) ^21^. This technology frees the user from several cables and thereby improves comfort for the patient. If the recording device has been planned to be self-applicable at home and is used at home during the night, having fewer cables reduces the number of failures and connection problems. Sometimes, a Bluetooth application can be used to send data to a smartphone or a tablet for signal quality control, online continuous monitoring, or other testing if required in a particular setting.

Data transmission from a recording device worn on the body of a patient or a volunteer to a remote monitoring center is part of telemedicine regulations and specifications. Telemedicine technologies had been developed for this purpose ^22^. Prototypes for this kind of application had been developed in the 1990s. One outstanding application is the continuous monitoring of vigilance in persons for whom high vigilance at their job is required. A system for online real-time monitoring of vigilance in situations similar to those of occupational medicine had been presented by Kim *et al*. ^23^. This particular system allows monitoring of EEG, EOG, and ECG in order to detect sleepiness and the onset of sleepiness in soldiers. For this purpose, six dry electrodes without any electrode gel were integrated in the helmet. These electrodes were used to record EEG, EOG, and ECG continuously. The EEG signal was simply processed to filter out just the alpha waves. The system used signal filtering for all electrophysiological signals with a band-pass for frequencies between 0.5 and 35 Hz. Then signals were digitized with 200 Hz. Data transmission of recorded signals was performed with Bluetooth technology. This portable helmet integrated system was validated against another conventional system for portable EEG recording with storage memory. As a result, the transmission of alpha waves, eye movements, and ECG went very well. The accuracy of the detection of sleepiness on the basis of these signals had a sensitivity of 79%, a specificity of 76%, and an agreement of 90% ^23^. The final applicability of the full system was limited because the sleepiness evaluation could not be performed in real-time or fully automated. Nevertheless, a telemetric assessment of vigilance is an emerging field of research and development. This is the case because several occupational areas, such as professional drivers and people doing supervisory tasks and similar jobs, really require continuous and real-time vigilance monitoring. Often such systems are subject to patents, and technologies are proprietary, and then the systems are published with considerable delays. Without any doubt, this field of applications is of immense economic importance because human failures due to low vigilance and prolonged or inadequate reactions of personnel can create enormous costs.

A full wireless recording of sleep, respiration, and heartbeat has been developed as well. The first sensor systems which used foils in the bed to monitor sleep were used in Finland. Alihanka and Vaahtoranta were among the first to use this technology based on electrostatic effects with pressure-sensitive foils underneath a bed blanket in order to record sleep, heart rate, and respiration during sleep without any contact with the patient being investigated ^24^. With an optimization of sensor technology, an improvement in signal-filtering methods, and the introduction of digital technology for signal processing and signal acquisition, it was possible to introduce an automatic analysis and diagnosis of sleep apnea in 1989 with additional information provided by the recording of oxygen saturation ^25^. This analysis has been validated against polysomnography. The technology for the recording of movements and their differentiated analysis has been further developed and improved in Finland. Today, very small and effective systems which make use of high-quality sensing and signal processing are available. Thereby, it is possible to record heart rate, sleep, and sleep apnea with high overall accuracy ^26^. With these new systems and similar ones, the hardware connects wirelessly to a recording system, such as a tablet or a smartphone, usually using Bluetooth. Also, the storage of data is wireless by making use of cloud-based storing. The analysis of the recorded data in terms of filtering and the detection of movement, respiration, and heart rate from these ballistocardiograms are performed by an app downloaded to a tablet or smartphone. As a consequence, the hardware costs (computing and storage) become low. The potential user needs to purchase the sensors only. The sensors can be regarded as consumables per test applied. The analysis software needs to be provided as an app and could be a low cost compared to the sensors. The application of these systems becomes very easy for the user ^27^. Then such systems will be available not only within the medical and physician-dominated field but also in the wellness and lifestyle market which serves general health assessment using internet marketing channels ^20^. The transition from medical diagnostic procedures to wellness products and the general “quantify yourself” movement becomes smooth and easy.

**Figure 2.**
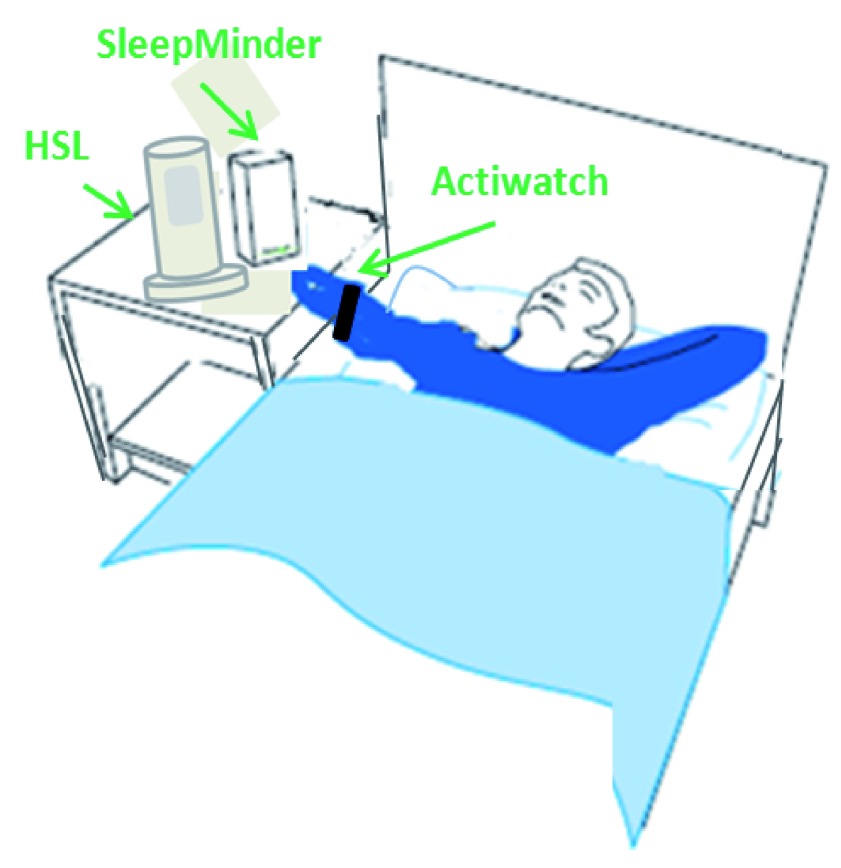
Non-obtrusive contact-free sleep recording with validated tools. Two systems for the contactless detection of respiration, body movements, and heartbeat are installed next to the bed of the sleeping subject ^29^. Both systems use Doppler technology with radar frequencies to detect the very small movements created by respiration and the heartbeat through a blanket. Larger movements are recorded as well and are taken as major body movements. A third and more traditional technology is tested in parallel. This is actigraphy with a wristwatch-like system. Actigraphy has been accepted as a non-invasive system to quantify sleep-wake times with acceptable accuracy, if being validated.

Also wireless, but using a different sensor approach, are systems which use another modern technology. These systems make use of electromagnetic wave reflections ^28^. Radio frequency waves, similar to radar technology, can detect very small body movements, as caused by heartbeat and respiration, even through the duvet of a sleeping subject ^28, 29^. Again, these reflection signals need to be filtered and processed. An optimized processing of the movement signals allows clinicians to distinguish sleep and wakefulness ^29^, sleep apnea, and normal breathing ^30^. These systems then allow the supervision of sleep in elderly persons in senior living houses or in hospitalized patients without much subject interference. Sleep-wake scheduling disorders can be recognized and quantified easily, and appropriate treatment (such as light treatment, movement stimulation, or social interaction planning) can be initiated early. In patients who are undergoing telemedicine monitoring owing to other reasons (for example, cardiac telemedicine monitoring because of heart failure or frequent cardiac arrhythmias), this wireless monitoring of sleep and breathing can be installed very easily in the subject’s home. In those patients with cardiac problems, it may be easy and justified to investigate the pattern of nocturnal breathing in order to detect “Cheyne–Stokes respiration”. This specific sleep-disordered breathing disorder with central apneas present is often found in patients with heart failure and has a negative predictive value for the survival of these patients. An early diagnosis of Cheyne–Stokes respiration and subsequent treatment of this condition will improve these patients’ quality of life. Moreover, guideline-oriented heart failure treatment together with a treatment of this respiratory condition improves life expectancy. In patients who cannot be investigated by using in-lab polysomnography because of other mental disorders or a medical condition, this kind of contactless sleep recording may also be a feasible option ( Figure 2).

## The smartphone tool – consumer and diagnostic apps

**Figure 3.**
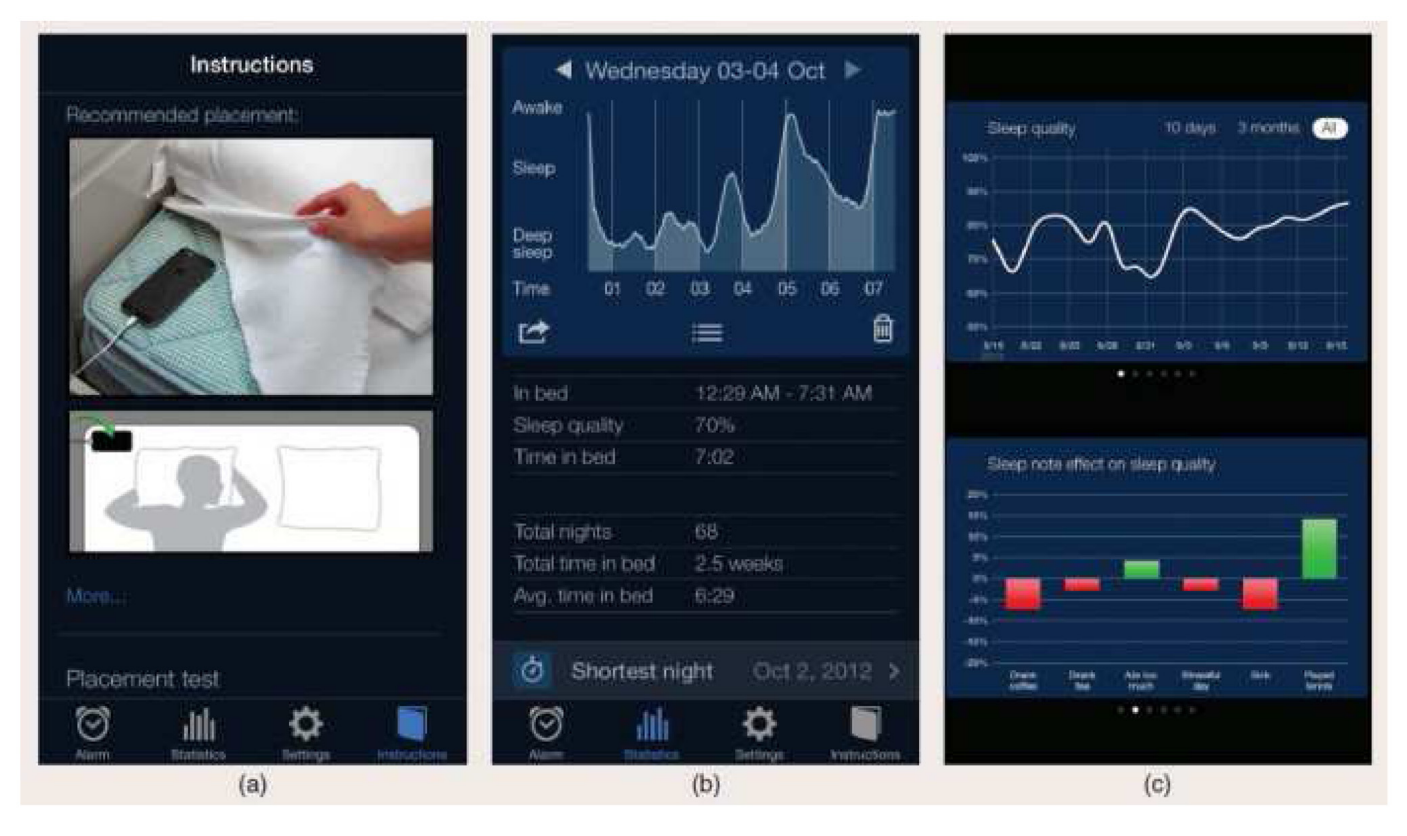
Smartphone applications to track sleep without external sensors. Smartphone applications use movement patterns in order to detect sleep, sleep disorders, and sometimes even sleep apnea ^21^. Here, we present the screenshots of a simple app which tracks sleep depth and, based on this, derives sleep duration and a kind of sleep quality. Frame ( **a**) shows how the smartphone should be used (placed underneath the blanket). Frame ( **b**) shows the report of a single night with a trace reflecting activity and estimating sleep depth. Frame ( **c**) shows the sequence of several nights summarized and with that a tracking of sleep quality over one week or a longer time period. Unfortunately, most sleep apps similar to this one have not been checked against a sleep recording or validated clinically and scientifically. They remain to be gimmicks for lifestyle use.

Today, smartphones are widely in use; indeed, in industrialized countries, every other person owns one. With each new generation of developments, new smartphones have more computational power and more and improved sensors. Making use of ambient light, ambient noise, accelerometer analysis to detect stationary (non-movement) patterns, and smartphone usage (lock and check phone) patterns allows clinicians to accurately estimate the owner’s wake and sleep behavior for up to 42 minutes ^31^. Future developments will allow additional external sensors to be added to assess more biological signals, especially in the consumer landscape ^20^. One promising approach to be used as a diagnostic tool is to add low-cost oximetry probes to a smartphone ^32^. Currently, this particular system is designed to provide an instantaneous oxygen saturation value together with a pulse rate value. For that purpose, validation results are extremely good ^32^. Sleep medicine applications, such as sleep apnea recognition, would need a continuous oximetry curve over a period of 8 hours instead of a single value. This would put additional demands on the system in terms of battery power, beat-to-beat signal processing, and graphics control. Beat-to-beat processing is important in order to maintain the rapid changes in oximetry which are very typical for sleep apnea and not observed in other conditions. Although this could be realized easily, such new hardware/software systems would require adequate validation. This long-term monitoring of oximetry with an external sensor and smartphone recordings, analysis, and display has been realized for screening children with sleep apnea and hypopnea ^33^. This device was validated against regular recording methodology. Another smartphone application uses external oximetry, respiratory effort by built-in microphone and movement by built-in accelerometer, to detect obstructive sleep apnea in adults ^34^. The sensitivity was 100% and the specificity was 85.7% in classifying obstructive sleep apnea in 15 participants ^34, 35^. Smartwatches go beyond the implementation of smartphones with external sensors. This is because smartwatches, worn on the wrist, are able to pick up bodily signals directly without additional sensors being attached. A smartwatch with light sensors at the bottom can record the pulse wave signal wherever the device is attached. Some people wear it on the wrist, others on the upper arm. The smartwatch gets the signal from the pulse wherever it is picked up. Then sophisticated algorithms extract heart rate and oxygen saturation. It is possible that a smartphone could use a wrist-worn smartwatch with optical light-emitting diode (LED) and other sensors as an external sensor feeding body signals to the smartphone with large memory and enough computational and battery power ( Figure 3).

Already more than 10 years ago, mobile phones were used for the diagnosis of sleep apnea ^36^. Researchers used external sensors to record a sleep EEG on the head with an application belt which was self-applicable. The consumer sleep tracker Zeo Sleep Manager Pro uses the same approach ^31^. Data recorded in this way may be transmitted to a smartphone again. This type of combined sensor and smartphone app may replace earlier portable sleep monitoring systems. Owing to the very high digital storage capacity (compared with early digital medical devices), it is possible to store all raw signal data. These signal data may be evaluated by using conventional visual scoring by trained medical sleep technicians or may be evaluated by using sleep analysis software. In this kind of setting, the smartphone serves as simply a mobile and easily available data logger with a large digital storage capacity and easy communication with external connected sensors. Evaluation and analysis of data would remain traditional with an established and accepted visual scoring methodology. Data need to be transferred to a sleep scoring center. One possible option, to transfer data to a sleep center, is wireless data transmission to upload data to cloud-based data storage. This data storage could be linked to a personal health database. This kind of cloud-based personal health-care database is under development by the medical informatics community in order to support the traveling patient. The traveling patient may grant access to all of his or her medical data by giving access to his or her cloud-stored health data to a physician at another location or country with adequate internet access. After completed scoring of sleep data by somebody who also has access to the cloud-stored data, the findings and reports could be stored in this personal health database, which then may serve as a personal electronic health record. Until now, the already-available personal health database records, more in the area of wellness and lifestyle, have contained activity data such as step counting, activity tracking, and entered food data only (supported by Google or iCloud services). From a data safety and health-care regulation viewpoint, there is ongoing discussion about which data have to be regarded as general wellness and lifestyle data and which data have to be regarded as health-care data to be used for medical diagnostic purposes. Currently, the line between the two areas is fuzzy. This is because low-cost sensors allow clinicians to access valuable data which can be useful for medical diagnosis as well. Many regulatory aspects are still open to this kind of application. This is because the currently applicable medical device certification focuses on devices and software in the hands of physicians and medical assistants. Now, as new devices (for example, a smartphone with a low-cost oximeter probe) make their way to the consumer, these consumer-acquired data may become medical data ^20^. Safety and security regulations for these kinds of data coming from a non-medical device may not have been tested for medical correctness and appropriateness. All of these technologies are summarized under the topic of “quantify yourself”. Definitely part of quantify yourself is the assessment of sleep. Now, how far the assessment of sleep may serve diagnostic purposes for sleep disorder assessment or how far it remains to be wellness feedback without any medical validation and without any diagnostic value is totally open and needs to be evaluated in carefully designed studies in the future.

## Sleep apnea treatment adherence monitoring

In many countries, worldwide telemedicine is used in sleep medicine to monitor therapy adherence in patients with sleep apnea using CPAP treatment ^17, 18^. For a number of years, many CPAP devices have had integrated digital storage to record patient usage data. In addition, CPAP devices, which regulate air pressure instantaneously, such as automatic titrating positive airway pressure devices, can continuously record the air pressure applied. Moreover, these devices can record air leakage and more signals. Based on this, the devices detect apnea, hypopnea events, and snoring periods. This detection is used to adjust the pressure applied. This detection can be used in a diagnostic mode to record apnea and hypopnea as well as flow limitation. A single-breath flow limitation pattern can be analyzed and saved for later inspection. All of these data can be stored in the device’s digital storage for years. Usually, only aggregated data for longer periods and few real-time data are saved. Data are downloaded and checked when a patient returns to a therapy follow-up study. The patient needs to take only the memory card to the sleep physician. CPAP devices are now equipped with simple global system for mobile communication (GSM) modules to transmit exactly these usage and aggregated diagnostic data to medical service providers. Who gets access to these data with personal usage information varies from country to country according to local medical data regulations. Therefore, there are many differences in the way sleep physicians get access to these data. The national sleep apnea management procedures are very different and involve medical service providers, health insurance, sleep centers, outpatient health care, and health data regulations. The German regulations are very restrictive and require a clear distinction of tasks among health insurance, service provider, and sleep physician ^18^. The American regulations require strong personal protection and transparency for data flow ^17^. In Australia and several European countries, the usage data are regularly transmitted to the service provider. The service provider receives money from the national health-care system only as long as the patient is using the device and only if the patient uses the device for long enough. If the usage decreases, it is up to the service provider to care for treatment adherence, and if the problem cannot be solved, the service provider has to refer the patient to a sleep center again. In some countries, there are service projects where a sleep physician gets direct access to the usage data of the patient and thus can get in contact with a patient where leakages, low usage time, and a high number of residual apnea events are observed. The physician can discuss potential problems with the patient and may provide consultations to solve the problems arising at an early point in time by improving therapy. Anonymized and aggregated user data can be evaluated with methods of big data statistics in order to identify risk profiles for good users and not-so-good users. It is possible to identify age- and gender-specific profiles and associations with concomitant disorders, co-morbidities, and specific usage patterns ^37^. The results of these analyses make it easier to consult patients from the beginning of the CPAP therapy and to advise on specific upcoming difficulties in therapy adherence. With this, it is possible to serve patients better, to address problems early, and to inform on adverse side effects as well. In the end, this improves patient satisfaction and treatment adherence. Whether these foreseen beneficial effects come true and are sustainable needs to be evaluated in large prospective trials using this new technology.

## Conclusions and perspectives

The most widespread application of telemedicine in sleep medicine relates to the recording of therapy compliance of CPAP in patients with sleep apnea. This technology has the potential to be used for non-invasive ventilation in other types of patients and in more advanced machines for respiratory support. Currently, this is being tested in small pilot projects. Patients with non-invasive ventilation require a much closer therapy control protocol compared with patients with sleep apnea. Therefore, continuous telemonitoring appears to be very useful in these patients. Telemonitoring may be useful in patients with sleep apnea who require other modes of therapy. A telemonitoring for maxillo-mandibular protrusion devices is considered. However, this requires sensor integration in these treatment devices. A possibility is temperature monitoring for the assessment of usage time. However, the remaining apnea events need to be assessed in a different way. For an implantable pacemaker (for example, a nervous hypoglossus stimulator or a nervous phrenicus stimulator), treatment monitoring is simple because it is already implemented in other cardiac pacemakers. Only regulatory hurdles and costs have to be considered here.

The exact rules regarding what a health service provider can do with the medical data need to be clarified in many countries. The usage time is the primary parameter for therapy adherence. The additional data, such as airflow shape, flow limitation information, and possible evaluation of remaining hypopnea and apnea events, need to be interpreted by a sleep physician in order to decide whether a change of therapy, such as an adjustment of applied pressure, is required. The communication with the CPAP device does not necessarily need to remain in the mode of monitoring usage and treatment. Instead, bidirectional communication can make use of changing the applied CPAP remotely. This is a useful feature for countries with large distances between patients and sleep centers, such as Australia. If such a feature is implemented, additional regulations need to be considered. Who is liable if the pressure adjustment is not functioning correctly? Is this a matter of data transmission, or was the device in use with a different subject? Before bidirectional telemedicine is implemented in the therapy of sleep apnea on a large scale, several technical and legal questions need to be clarified.

Much less problematic is the application of telemedicine in the area of diagnosis for sleep apnea and sleep disorders. Patients definitely will profit from improved comfort using fewer wires and being recorded more often at home or in their natural sleep environment. Here, telemedicine is a more modern technology for data transmission. One needs to consider whether the technology should be used to record over a distance, reduce the number of wires on the body itself, allow data transfer to central data servers, or transfer diagnosis on a smartphone with integrated and additional external sensors. The questions to be solved here are mainly questions for data safety and data security and questions on the management of sleep disorders. A discussion is needed as to what extent sleep recording and the diagnosis of sleep disorders constitute medical management or lifestyle management ^20^. Which sleep disorders and which severities of sleep disorders should remain in the area of wellness and lifestyle and which should be in the hands of sleep medicine and sleep physicians? For the current use of existing sleep apps in smartphones, it is most important to inform users and patients that only a few have been validated against polysomnography. Not only users but also physicians need to be informed about the potential usefulness and limitations of sleep apps as they become available. This is needed because, already, many patients come after having used smartphone apps with reports on their sleep quality. Patients come to sleep physicians with the results provided by a smartphone sleep app and are worried if the app reports that their sleep was bad. The sleep app may propose a medical diagnosis (for example, insomnia in the case of short sleep or sleep apnea in the case of observed respiratory cessations) and the patient then asks for treatment or a consultation based on this. Information and education are required to address this issue in the broader public.

## Abbreviations

AHI, apnea hypopnea index; CPAP, continuous positive airway pressure; HSAT, home sleep apnea testing; PG, polygraphy; PSG, polysomnography; RIP, respiratory inductive plethysmography.
